# Quality of maternity care provided by private sector healthcare facilities in three states of India: a situational analysis

**DOI:** 10.1186/s12913-019-4782-x

**Published:** 2019-12-16

**Authors:** Sanjay Tripathi, Ashish Srivastava, Parvez Memon, Tapas Sadasivan Nair, Parag Bhamare, Dinesh Singh, Vineet Srivastava

**Affiliations:** 1 Jhpiego an affiliate of Johns Hopkins University, Lucknow, Uttar Pradesh India; 2 Jhpiego - an affiliate of Johns Hopkins University, Jhpiego, 29, Okhla Phase 3, New Delhi, India; 3 Jhpiego - an affiliate of Johns Hopkins University, Mumbai, Maharashtra India; 4 Jhpiego - an affiliate of Johns Hopkins University, Ranchi, Jharkhand India

**Keywords:** Quality of care, Facility preparedness, Private sector, Intrapartum care, Postpartum care, Maternal health, Quality improvement

## Abstract

**Background:**

Better quality of care around the time of childbirth can significantly improve maternal and newborn survival. In countries like India, where the private sector contributes to a considerable proportion of institutional deliveries, it is important to assess the quality of maternity care offered by private sector healthcare facilities. This study seeks to fill that information gap by analysing baseline assessments conducted for the Manyata program, which aims to improve the quality of maternity care at private facilities.

**Methods:**

An observation checklist based on 16 clinical standards endorsed by the Federation of Obstetric and Gynaecological Societies of India (FOGSI) was used to assess 201 private sector healthcare facilities in Maharashtra, Jharkhand, and Uttar Pradesh. Data on facility characteristics came from profiles completed when facilities enrolled in Manyata. Differences in the mean number of standards met were analysed by facility characteristics and the availability of essential supplies.

**Results:**

Around half (47.1%) of all nursing staff engaged in maternity care services at these private healthcare facilities were under qualified. The mean number of clinical standards met by facilities was 3.2 (SD 2.4). Facilities with a monthly delivery load between 20 and 50 met a significantly higher number of standards, as did facilities that had more than 70% of essential supplies available. Both these factors were also significant in a multiple linear regression analysis.

**Conclusions:**

The overall quality of maternity care in private healthcare facilities is poor in all three states, especially for clinical standards related to management of complications.

## Background

Improving quality of care is essential to ensure patient safety and accelerate reductions in mortality and morbidity [1]. To prevent avoidable maternal and neonatal deaths, every pregnant woman and newborn baby needs skilled care at the time of birth, with evidence-based clinical and non-clinical interventions delivered in a compassionate and enabling environment [2, 3]. Global evidence has proven that better quality of care at childbirth could avert up to 1.49 million maternal and newborn deaths and stillbirths annually and significantly improve maternal and newborn survival [4, 5]. The majority of maternal deaths (over 70%) result from complications that require facility-based care, such as postpartum hemorrhage, hypertensive disorders, sepsis, and complications related to abortions [6]. Therefore, improving the quality of facility-based delivery care offers tremendous opportunities to reduce maternal and perinatal deaths [7].

In India, after the launch of government’s conditional cash transfer scheme for promoting institutional deliveries, the Janani Suraksha Yojana (JSY), rate of institutional deliveries has nearly doubled over the last decade [8]. However, the significant gains achieved in maternal and neonatal mortality were not sufficient to help the country achieve Millennium Development Goals 3 and 4 [9]. Therefore in the context of Sustainable Development Goal 3 [10], it is essential to focus on the provision of high quality care during childbirth to reduce adverse maternal and neonatal outcomes [11, 12]. This requires a focus on private as well as public sector healthcare facilities. The private sector contributes to a considerable proportion of institutional deliveries across the world [13] and plays an important role in delivering health care services in India, providing 80% of all outpatient care and up to 60% of inpatient care. As many as 60% of hospital beds in India are in the private sector, as are the majority of human resources, including 70% of the total health workforce, 80% of physicians, and most obstetricians [14]. According to the National Family Health Survey 4 (NFHS-4), the private sector accounts for up to 22% of institutional deliveries in rural areas and up to 43% of institutional deliveries in urban areas [15].

Although the Government of India has introduced several initiatives targeting various aspects of quality in service delivery and facility operations of public sector health facilities [16–18], the private sector has not received much attention. The bulk of available research evidence on the quality of essential care at the time of birth – mostly from public sector facilities in low- and middle-income countries (LMICs) – highlights the need to carefully examine and address deficiencies in the quality of care at the time of birth. However, research on the quality of maternity care in private healthcare facilities in India is limited [19]. In this context, it becomes important to understand the landscape of quality of maternity care in the private sector, make a systematic effort to measure, and identify quality gaps.

The Manyata program is a quality improvement and assurance initiative for private sector maternity care facilities across three states (Uttar Pradesh, Jharkhand, and Maharashtra) [20]. Facilities, which voluntarily opt, to participate in the program receive three days nurses training on skills and competencies related to key lifesaving practices and six deliverable linked mentoring support visits for six months period. Program also ascertains the quality of maternity care, once the facility completes its quality improvement journey and achieves a set number of standards at the final assessment to obtain Manyata certification, which is a seal of quality assurance. The program is being implemented by Jhpiego, an affiliate of Johns Hopkins University, with support from MSD for Mothers and in collaboration with the Federation of Obstetric and Gynecological Societies of India (FOGSI). Baseline assessments conducted under Manyata program provided an opportunity to measure the existing quality of care at childbirth in participating private healthcare facilities. This paper is a retrospective examination of the baseline data intended to yield insights on the quality of delivery care practices at private healthcare facilities in three states of India.

## Methods

### Study settings, study design and sampling

A structured checklist was used to assess 201 private sector healthcare facilities that offer obstetric care in 24 districts across the states of Maharashtra, Jharkhand and Uttar Pradesh. Uttar Pradesh is the most populous state in India with a population of 199 million. Maharashtra and Jharkhand are 2nd and 13th most populous states in India (out of 28 states) with a population of 112 million and 33 million respectively [21]. With respect to performance on maternal health, while Uttar Pradesh and Jharkhand are poor performing states with maternal mortality ratio (MMR) of 165 and 201 respectively, Maharashtra is a better performing state with MMR of 61 [22].

Baseline assessments for the Manyata program at these facilities were conducted from November 2016 to March 2017 (year 1 facilities) and from December 2017 to March 2018 (year 2 facilities). The criteria for including private healthcare facilities in the Manyata program are that the facility: 1) is registered with local health authorities, 2) provides maternity services, 3) has an owner or in-charge who is a member of FOGSI, and 4) expresses willingness to participate in the Manyata program by paying a nominal fee and submitting letter of intent to FOGSI.

### Study tool

**Fig. 1:**
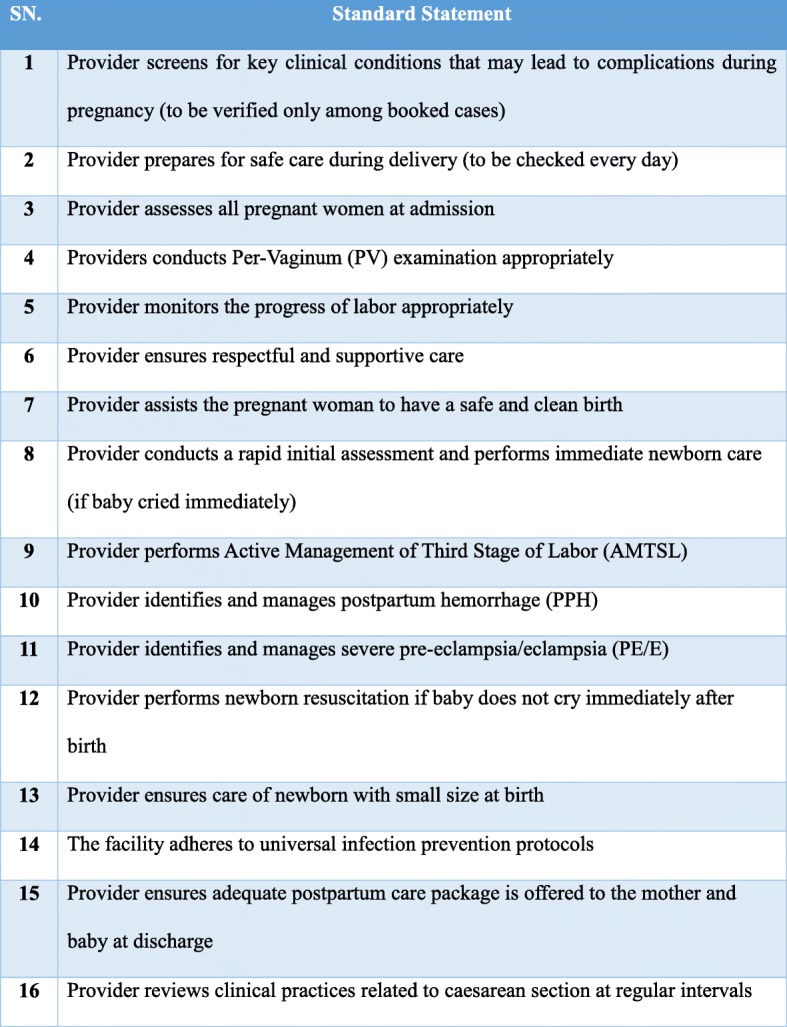
FOGSI Clinical Standards for Manyata Program

The assessment checklist was based on the standards for improving quality of maternal and newborn care in health facilities by WHO and endorsed by FOGSI [23, 24]. It includes 16 clinical standards that focus on: patient care during the antenatal period (one standard), the intrapartum period (13 standards), the postpartum period (one standard), and caesarean section (one standard). Each standard includes four or five essential elements and has five or six verification criteria, which ensure the objective assessment of providers’ skills and knowledge (Fig. 1).

Assessors recorded **“Y”** for each verification criterion that the provider or facility met and **“N”** for unmet criteria. If all of the verification criteria listed under a standard were met, the standard was considered as met and given a score of 1**.** If any verification criteria were **not** met, the entire standard was considered unmet and given a score of zero**.** The facility score was calculated as the total number of standards met; the highest possible score was 16.

### Data collection

Manyata Program Officers, who were either nursing professionals or doctors, conducted the baseline assessments. They were oriented and trained on the assessment methodology and technical components of the standards as part of the program’s Training of Trainers (ToT). These three-day ToTs were conducted by the program’s clinical team, which consisted of senior obstetricians with public health experience. Assessments at each facility were usually spread across two days and required 4–6 working hours per day, using a mixed methods approach.

Each verification criterion was assessed using one of the following four methods: 1) direct observation of service providers during skills demonstration on mannequins or during provision of actual care; 2) hospital record reviews to check for the implementation of practices; 3) provider interviews to assess knowledge; or 4) physical verification of the presence of drugs, supplies, functional equipment, and instruments in the labor room. Verification criteria related to provider skills were assessed through observations, those related to provider knowledge were assessed through interviews, those related to routine practices at the facility were assessed through record reviews, and those related to availability of drugs and equipment were assessed through physical verification.

In addition, information on facility characteristics, such as facility type based on the availability of services, average monthly delivery load, availability of human resources, and number of hospital beds, were collected when the facility registered for the Manyata program. For availability of human resources, data on number of qualified nursing staff per facility was collected. Qualified nursing staff refers to those nursing personnel who have undergone formal training under any of the Indian Nursing Council prescribed programs [25].

### Data analysis

For the purpose of this analysis, a separate dataset was created by combining the baseline assessment data with the facility characteristics data. Data were cleaned and checked for completeness. We computed the mean number of standards met by the facilities, the proportion of facilities that met each individual standard, and the proportion of facilities that met a set number of standards. We analyzed variations in the mean number of standards met by various facility characteristics and the availability of essential supplies. To identify determinants of the number of standards met by a facility, we conducted a multivariable linear regression analysis, with number of standards met by facilities as the dependent variable and facility characteristics and availability of essential supplies as independent variables. We used independent sample t test and ANOVA for comparing means of two and three subgroups respectively. A *p*-value of less than 0.05 was considered as statistically significant. Statistical Package for the Social Sciences (SPSS), version 24, were used to carry out the data analysis.

## Results

### Characteristics of facilities

Two-thirds of the 201 private healthcare facilities enrolled in the Manyata program were from Uttar Pradesh and Jharkhand collectively (Table 1). A majority (87%) were small health care providers with fewer than 50 functional beds. The average monthly delivery load for most facilities (59.7%) was less than 20 deliveries. Most of the facilities (87%) had at least one qualified nurse or midwife on staff, although almost half (47.1%) of the nursing staff at the facilities were under qualified (data not shown).

**Table 1:** Characteristics of private health care facilities (n = 201)

Characteristics	Number	Percent
State		
Uttar Pradesh	101	50.3
Maharashtra	67	33.3
Jharkhand	33	16.4
Number of hospital beds		
≤ 50	184	91.5
> 50	17	8.5
Monthly delivery load		
< 20	120	59.7
20–50	62	30.8
> 50	19	9.5
Type of facility		
Exclusive maternity hospital	112	55.7
Multispecialty hospital	89	44.3
Availability of qualified nurse or midwife		
None on staff	26	12.9
At least one on staff	175	87.1

### Standards met

The mean number of standards met by private health facilities across the three states was 3.2 (SD 2.4). Less than one-fourth (24.4%) of the 201 private healthcare facilities met more than four standards (Table 2).

**Table 2:** Distribution of facilities according to number of standards met (n = 201)

Facility score (number of standards met)	Number of facilities	Percent
0–4	152	75.6
5–8	41	20.4
9–12	7	3.5
13–16	1	0.5

**Fig. 2:**
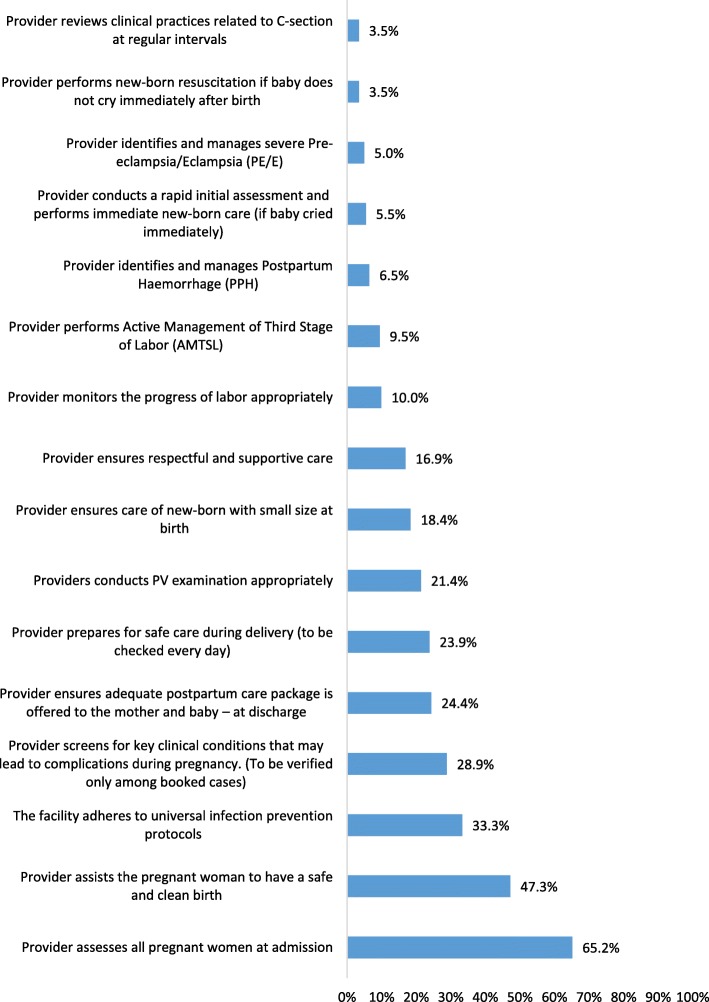
Proportion of facilities that met each standard (*n* = 201)

The standard most often met, by 65.2% of facilities, was assessing pregnant women at admission. Standards for newborn resuscitation and periodic review of clinical practices related to caesarean section were the least often met (3.5%) (Fig. 2). Only 17% of facilities met the standard for respectful and supportive care, mostly because facilities failed to allow a birth companion during labor.

The mean number of standards met varied across states, with Uttar Pradesh having the highest mean score (3.61) and Jharkhand the lowest (2.76), but this difference was not statistically significant. However, mean scores did vary significantly by monthly delivery load and availability of essential supplies. The mean number of standards met by facilities with 20–50 monthly deliveries was significantly higher than other facilities (4.14 versus 2.97 and 2.42). Facilities that had more than 70% of essential supplies also met significantly more standards than other facilities (4.54 versus 1.96) (Table 3).

**Table 3:** Mean number of standards met, by facility characteristics (n = 201)

Characteristics	Mean number of standards met (SD)	*p* value^#^
State		
Uttar Pradesh	3.61 (SD 3.3)	*P* > 0.05
Maharashtra	3.42 (SD 2.1)	
Jharkhand	2.76 (SD 2.2)	
Number of hospital beds		
≤ 50	3.26 (SD 2.4)	P > 0.05
> 50	2.94 (SD 2.9)	
Monthly delivery load		
< 20	2.97 (SD 2.1)	*P* < 0.05
20–50	4.14 (SD 2.9)*	
> 50	2.42 (SD 2.1)	
Type of facility		
Exclusive maternity hospital	3.36 (SD-2.4)	P > 0.05
Multispecialty hospital	3.07 (SD- 2.4)	
Availability of qualified nurse or midwife		
None staff	2.92 (SD 1.4)	P > 0.05
At least one on staff	3.27 (SD 2.5)	
Availability of essential supplies		
≤ 25 items (70% of supplies)	1.96 (SD 1.6)	P < 0.05
> 25 items (70% of supplies)	4.54 (SD 2.4)	

# Estimated using independent sample t test for two sub groups and ANOVA for more than two sub groups, ^a^ varied significantly with other two categories on using post-hoc test

### Determinants of number of standards met

The multivariate linear regression analysis found that monthly delivery load and availability of essential supplies were significant determinants of the number of standards met by the private healthcare facilities. Facilities with a moderate delivery load (20–50 deliveries per month) and facilities with at least 70% of essential supplies available met a significantly more standards (Table 4).

**Table 4:** Multiple linear regression analysis on number of standards met by the facilities

Variable	Standardized coefficient (S.E.)	95% confidence interval
State		
Jharkhand	Reference category	
Maharashtra	- 0.44 (0.48)	−1.39 – 0.51
Uttar Pradesh	0.37 (0.47)	−0.55 – 1.30
Type of facility		
Multispecialty hospital	Reference category	
Exclusive maternity hospital	0.31 (0.34)	−0.37 - 0.99
Number of hospital beds		
≤ 50	Reference category	
> 50	0.09 (0.58)	−1.05 - 1.24
Monthly delivery load		
< 20	Reference category	
20–50	0.72 (0.35)	0.02 - 1.41*
> 50	−0.24 (0.54)	−1.32 - 0.82
Availability of essential supplies		
≤ 25 items (70% of supplies)	Reference category	
> 25 items (70% of supplies)	2.49 (0.31)	1.86 - 3.12*
Availability of qualified nurse or midwife		
None on staff	Reference category	
At least one on staff	0.54 (0.48)	−0.41 - 1.51

*p < 0.05

## Discussion

Overall, the quality of care around childbirth at private healthcare facilities was poor. Three-fourths of facilities enrolled in the Manyata program met only four or fewer of the 16 FOGSI-endorsed clinical standards; only one facility met more than 12 of the standards. This is similar to findings from the few other studies carried out in Uttar Pradesh and Maharashtra that have evaluated the quality of care in private healthcare facilities [26–28]. However, the results are noteworthy as private health care facilities are generally perceived to be more efficient and provide better quality of services [29]. This is probably the reason why Indian families who can afford to pay prefer private sector services over the public health system, despite the added expense [30].

One reason why the quality of care may be so poor at these private sector healthcare facilities is the lack of qualified staff. We found that the mean number of standards met was higher at facilities with at least one qualified nurse or midwife on staff, although this difference was not statistically significant. Almost half of all nursing staff engaged in maternity care services at these private healthcare facilities were under qualified, and 13% of facilities did not have a single qualified nurse or midwife on staff. These findings are similar to a previous analysis by Rao et al [31], which estimated that 58.4% of nurses and midwives serving in the private healthcare facilities of India were under qualified. Likewise, a 2001 survey of private health care providers in Delhi [32] found that 41% were under qualified. In the absence of regulatory requirements for staffing, private facilities may employ unskilled staff to increase profit margins and compromise quality in the process [33].

Clinical care processes in private healthcare facilities in India are largely individually driven, as the government does not mandate the uniform use of standard care practices in the private sector. This heightens the importance of professional organizations like FOGSI in standardizing care practices, as they can play a critical role in reviewing and prescribing care protocols to members [34].

The baseline assessment found that standards related to the assessment of pregnant women on admission and assisting pregnant women to have a safe and clean birth were met by relatively more facilities, while standards related to managing complications – which are relatively rare – were met by very few facilities. This needs to be seen in the context of the assessment methodology, which considered the facility team (clinician and nursing staff) as a unit of measurement in assessing adherence to standards of care. Nursing staff are fully involved in routine care and thus could demonstrate their capabilities during the assessment. However, management of complications is heavily dependent on specialists at the facilities with limited involvement of nursing staff. This may be why the team could not demonstrate adherence to standards on management of complications.

Notably, the standard for respectful and supportive care was met by just one in six private healthcare facilities. Respectful care is a key component of quality of care, and mistreatment and poor quality of clinical care are closely interlinked [35]. The poor adherence to respectful care further correlates with existing evidence in both high [36–40] and low income settings [41–43], but contradicts the widespread perception that private healthcare facilities are more likely to provide respectful and supportive care due to their customer service orientation and concerns that a negative reputation on this front could hamper their profits.

A deeper look into the data revealed that the main reason why facilities did not meet the standard for respectful and supportive care was because of their failure to allow a birth companion during labor, which was one of the verification criteria for this standard. This hesitation in allowing a companion in the labor room may stem from fear of interference. Also, it is likely that many providers are not aware of the benefits of this practice for maternity outcomes [44].

Delivery load was a significant determinant of quality of care in the multiple linear regression; facilities with a moderate delivery load, between 20 and 50 deliveries per month, met significantly more standards than facilities with either lower or higher delivery loads. This load may be optimal because it ensures that staff receive regular practice but, at the same time, are not overburdened. Patient load and time spent with each patient by private providers have a significant bearing on the quality of health care [44]. The other significant factor in the regression was the availability of at least 70% of the essential items required for carrying out recommended practices. This corroborates previous studies [42, 43] that have found adequate supplies and infrastructure are important determinants of the quality of care in private sector healthcare facilities.

Among the three states where the Manyata program has been implemented, Uttar Pradesh and Jharkhand perform more poorly than Maharashtra on social and health indicators. Uttar Pradesh and Jharkhand score considerably below the national average of 0.639 on the Human Development Index [45], while Maharashtra scores above the national average. In addition, the private sector’s contribution to institutional deliveries is greater in Maharashtra (45.8%) as compared to Uttar Pradesh (34.4%) and Jharkhand (32.5%) [46–48]. However, this assessment found that the quality of care in private healthcare facilities was poor across all three states and did not vary significantly between states. This is corroborated by existing literature on the quality of care in private healthcare facilities in states like Maharashtra that found poor standards of care in small private health care facilities [26].

## Strengths and limitations

Our analysis is an important addition to the scarce literature on the quality of maternity care in the private healthcare facilities of India. The fact that the facilities assessed came from both higher (Maharashtra) and lower performing states (Uttar Pradesh and Jharkhand) in terms of key maternal health indicators is a major strength of this study. In addition, the assessment standards are based on WHO standards for improving quality of maternal and newborn care in health facilities and endorsed by FOGSI. Therefore, the findings will be comparable with future studies that use similar standards and approach. However, there are some limitations to the interpretation of the findings. The facilities included in the analysis had voluntarily opted to participate in the Manyata program, so the sample may not be truly representative of private healthcare facilities across the three states. Facilities voluntarily opting to participate in the Manyata program for getting Manyata certified may differ from general private health care facilities in terms of being more conscious about importance of quality parameters or standards. In addition, direct observations of providers’ skills were an important component of the assessment methodology and therefore, the findings are liable to the Hawthorne effect [49] that is health service providers may have been conscious of being observed and therefore improved their practices.

## Conclusion

To our knowledge, such an extensive situational analysis of the readiness of private sector healthcare facilities to provide good quality intrapartum and immediate postpartum care has not been done before in India. The Manyata program provided an opportunity to objectively measure the quality of maternity care in private sector healthcare facilities in terms of FOGSI-endorsed standards. This secondary analysis of program data is valuable to stakeholders across the public health community of India as well as in similar settings globally; these include government, policy makers, donors, and implementing organizations as well as the private sector healthcare facilities themselves.

Historically, health programs and policies have been designed mostly to address the needs of public sector health facilities, and it has been assumed that health care practices are better at private sector facilities. In contrast, this study places a spotlight on deficiencies in the quality of care at private healthcare facilities and provides important insights for building a common policy framework to ensure standardized care across both public and private sectors or for creating a policy specific to the private sector.

## Data Availability

Data are available from Jhpiego’s internal institutional data access committee for researchers who meet the criteria for access to data. Corresponding author can be contacted for further communication.

## Abbreviations

AMTSL: Active management of third stage of labor
ANOVA: Analysis of variance
FOGSI: Federation of Obstetric and Gynaecological Societies of India
MSD: Merck Sharp and Dohme
NFHS: National Family Health Survey
PE/E: Pre-eclampsia/eclampsia
PV: Per vaginum
SD: Standard deviation
SPSS: Statistical Package for the Social Sciences
ToTs: Training of trainers
WHO: World Health Organization; LMIC: Low and Middle Income Country
